# BRASH Syndrome Leading to Cardiac Arrest and Diffuse Anoxic Brain Injury: An Underdiagnosed Entity

**DOI:** 10.7759/cureus.18628

**Published:** 2021-10-09

**Authors:** Ghulam Mujtaba Ghumman, Aakash Kumar

**Affiliations:** 1 Internal Medicine, St. Vincent Mercy Medical Center, Toledo, USA

**Keywords:** ecg (electrocardiogram), cardiac arrest, hyperkalemia, av nodal blocking medications, renal failure, bradycardia, brash

## Abstract

BRASH (bradycardia, renal failure, atrioventricular [AV] nodal blocking medications, shock, hyperkalemia) syndrome describes the phenomenon of profound bradycardia from a combination of hyperkalemia and use of AV nodal blocking medication with underlying renal injury. We present a case of BRASH syndrome in a patient on chronic beta-blocker therapy for his coronary artery disease who presented with life-threatening hyperkalemia and acute renal failure. Due to failure in early recognition and superimposed effect with further beta-blocker dosing, the patient developed profound bradycardia and later went into pulseless electrical activity cardiac arrest requiring cardiopulmonary resuscitation. Metabolic derangements and bradycardia later resolved with medical management, but unfortunately, the patient developed diffuse anoxic brain injury after the cardiac arrest and was declared brain dead.

## Introduction

The BRASH (bradycardia, renal failure, atrioventricular [AV] nodal blocking medications, shock, hyperkalemia) syndrome is a pathophysiologic phenomenon comprising bradycardia, renal failure, AV blockade, shock, and hyperkalemia. It is an underdiagnosed entity that is usually treated but instead goes unnoticed. It was first described by Joshua D. Farkas in 2016 [1]. It is a vicious cycle consisting of bradycardia that develops from a combination of hyperkalemia and AV nodal blocking medications along with renal failure that itself can lead to hyperkalemia and delayed clearance of AV nodal blocking medications. Patients ultimately develop shock (poor organ perfusion) with further kidney damage that worsens the hyperkalemia, and thus bradycardia and the vicious cycle continue until intervened [2]. These patients usually develop renal failure from either dehydration or nephrotoxic medications, e.g., angiotensin-converting enzyme inhibitors (ACEI)/angiotensin II receptor blockers (ARBs), but the etiology could be any. Hyperkalemia develops directly from renal failure or potassium-sparing medications (ACEI/ARBs or aldosterone antagonists) [3]. These patients usually are on chronic AV nodal blocking medications (beta-blockers or calcium channel blockers), which along with hyperkalemia can lead to profound symptomatic bradycardia that can even lead to shock and cardiac arrest. If no intervention is done, shock can worsen the kidney function with the vicious cycle of worsening hyperkalemia and thus bradycardia [1].

## Case presentation

**Figure 1:**
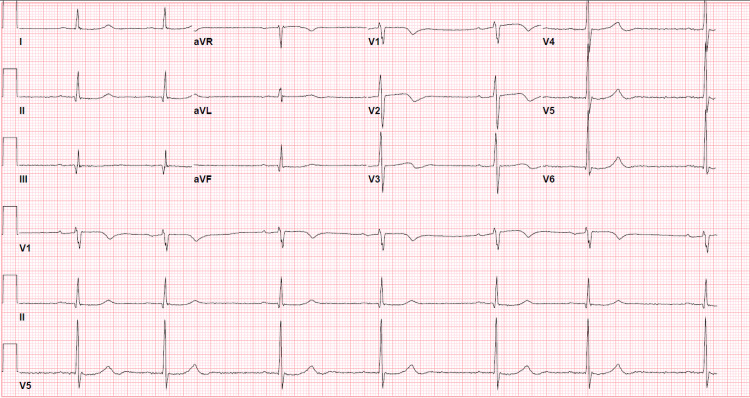
ECG shows sinus bradycardia with first-degree atrioventricular block

We present a case of a 69-year-old male who has a past medical history of atherosclerotic coronary artery disease status post percutaneous transluminal coronary angioplasty with a drug-eluting stent in 2018, essential hypertension, and insulin-dependent type-II diabetes mellitus. Home medications included metoprolol succinate, lisinopril-hydrochlorothiazide, aspirin, clopidogrel, rosuvastatin, metformin, and insulin Degludec. The patient was brought to the emergency department at an outlying facility by Emergency Medical Services when the family could not wake him up from sleep during the afternoon nap. The patient exhibited Cheyne-Stokes breathing and was deeply obtunded on presentation. He was intubated to protect the airway because of the low Glasgow Coma Scale score of 5. The patient was in a state of hypertensive emergency with a blood pressure of 253/124 mmHg. The patient received 20 mg of labetalol for high blood pressure, and shortly after, he started to become bradycardic and later went into pulseless electrical activity. Cardiopulmonary resuscitation (CPR) was started, and return of spontaneous circulation (ROSC) was achieved after 10 min of CPR with three rounds of epinephrine and 1 g of calcium chloride. Initial laboratory workup showed that the patient had acute kidney injury (AKI) (creatinine 2.14 mg/dL with baseline creatinine in the normal range), severe hyperkalemia (potassium 7.9 mmol/L), bicarbonate 21 mmol/L, lactate 4.7 mmol/L, high-sensitivity troponins 20 ng/L (0-22 ng/L), blood glucose of 550 mg/dL, beta-hydroxybutyrate 0.32 (0.02-0.27 mmol/L), and negative urinary ketones. Intravenous (IV) calcium gluconate, insulin, and dextrose were administered for hyperkalemia. After the ROSC, the patient remained hypotensive and bradycardia with a heart rate in the 30s (beats per min); he was given IV fluid blouses, and epinephrine infusion was started. Initial ECG showed sinus bradycardia with first-degree AV block without other electrocardiographic features of hyperkalemia (Figure 1).

**Figure 2:**
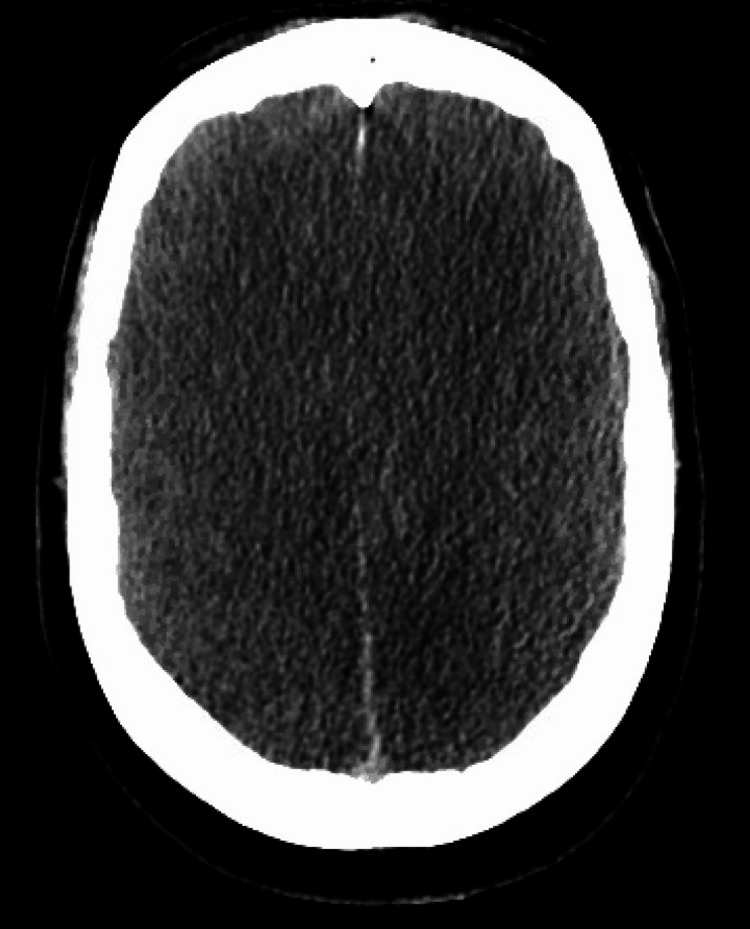
Axial CT scan of the head shows complete loss of gray-white matter differentiation and complete obliteration of brain sulci and cisterns suggesting diffuse anoxic brain injury CT, computerized tomography

**Figure 3:**
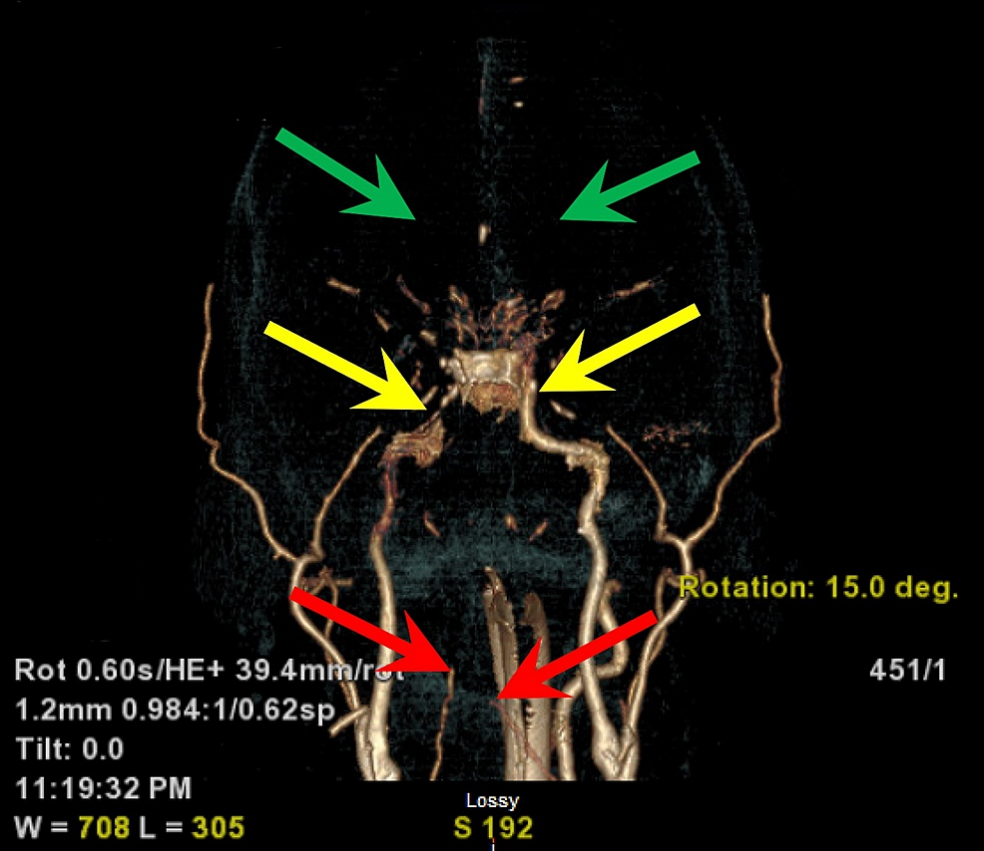
Reconstructed image of the CTA head and neck shows complete occlusion of bilateral vertebral arteries (red arrows) and complete occlusion of bilateral internal carotid arteries (yellow arrows) along with lack of intracranial circulation (green arrows) CTA, computerized tomography angiography

The patient was then transferred to our facility for a higher level of care. On arrival at our facility, the patient was already intubated but not requiring any sedation as he was not responsive, although some myoclonic jerks were noted. He was given a dose of 1 mg atropine and 5 mg glucagon for bradycardia with subsequent improvement of heart rate to 50-60 beats per min. The patient was started on targeted temperature management, and the patient’s body temperature was maintained at 33 degrees Celsius for 24 hours with gradual rewarming afterward. Hyperkalemia resolved with regular insulin and dextrose treatment. AKI resolved with IV fluid hydration. The patient was not on any sedation but remained unresponsive with absent corneal, pupillary, cough, and gag reflexes. Computerized tomography (CT) scan of the head and CT angiography scan of head and neck showed signs of diffuse anoxic brain injury and complete occlusion of all large vessels within the head and neck (Figures 2, 3).

Unfortunately, the patient was confirmed brain dead with a positive apnea test and absence of brainstem reflexes. The patient’s presentation and hospital course were suggestive of BRASH disease: bradycardia from hyperkalemia and AV nodal blocking medication with subsequent development of cardiogenic shock. The patient already was in AKI with hyperkalemia, which along with the effect of labetalol led to profound bradycardia and cardiac arrest. 

## Discussion

Joshua D. Farkas observed a phenomenon in 2016 where a combination of hyperkalemia and AV nodal blockade led to profound bradycardia in patients who had secondary renal insufficiency [1]. The phenomenon is now known as the BRASH phenomenon or BRASH syndrome. The BRASH phenomenon has been observed to occur with the development of bradycardia from the synergistic effect of hyperkalemia and AV nodal blockade that breaks into a vicious cycle with underlying kidney insufficiency [4,5]. These patients develop hyperkalemia from renal injury (due to poor renal secretion of potassium) and/or potassium-sparing medications (e.g., ACEI/ARBs or aldosterone antagonists) [6-8]. Renal injury develops from poor perfusion related to dehydration (hypovolemia) or nephrotoxic medications (e.g., ACEI/ARBs or digitalis) but could be from any etiology [9]. These patients are chronically taking AV nodal blocking medications (beta-blockers or calcium channel blockers), and their clearance can also be delayed because of renal injury leading to accumulation of their levels during the BRASH phenomenon. Beta-blockers can directly cause hyperkalemia, a phenomenon observed mainly with non-selective beta-blockers (e.g., labetalol, carvedilol) [10]. The synergism between AV nodal blocking medications and hyperkalemia leads to bradycardia, which gradually leads to hypotension (by decreasing the cardiac output) [11,12]. This leads to organ hypoperfusion, mainly of kidneys, further worsening the renal function and thus leading to the vicious cycle of renal failure - hyperkalemia - bradycardia - shock - renal failure [1,13]. This process continues to cause multiorgan dysfunction until intervention from outside to correct the kidney function. The mainstay of treatment is supportive therapy with fluid resuscitation to correct the renal function and therapies for hyperkalemia to break the vicious cycle causing bradycardia [14]. Patients might also need additional treatment for bradycardia with pacing or atropine [15]. It is important to recognize this process early in the disease course as early treatment leads to a good outcome.

It is clear from the literature that both hyperkalemia and AV nodal blockade can individually lead to bradycardia, but in BRASH, it is the synergistic effect of both hyperkalemia and AV node blockade that leads to profound bradycardia and thus multiorgan dysfunction. Distinguishing BRASH from "isolated hyperkalemia" and "isolated overdose of an AV nodal blocking medication" is vital. Isolated hyperkalemia can cause bradycardia and renal failure, but usually, bradycardia does not develop until the degree of hyperkalemia is severe. However, it is important to remember that severe hyperkalemia can develop from BRASH syndrome itself, as in our case. The hyperkalemia associated with BRASH syndrome can cause bradycardia but usually lacks other ECG features of hyperkalemia (tall, peaked T-waves and shortened QT interval followed by lengthening of PR interval, wide QRS, and disappearance of P-waves if hyperkalemia gets more severe), and that can be considered an important clue toward the diagnosis of BRASH syndrome [1,16]. Overdose of AV nodal blocking medications (e.g., beta-blockers, calcium channel blocker, or digitalis intoxication) suppresses conduction of electrical signals from atria to ventricles leading to bradycardia, which can cause shock and renal failure [5,11]. The AV nodal blockade in BRASH occurs not because of AV nodal blocking agents' overdose but rather due to the synergistic effect of AV node blockade and hyperkalemia. BRASH patients are taking their medications as prescribed to them, and AV nodal blocking medication level is therapeutic in the blood. Also, it is important to note that BRASH patients would have concomitant hyperkalemia [1].

## Conclusions

The BRASH syndrome is an underdiagnosed entity that is usually unnoticeably treated by physicians. The literature review shows few cases of BRASH syndrome, and it is gaining more awareness among physicians. The treatment of the BRASH is simple, and outcomes are usually good. We emphasize the importance of recognizing and treating this phenomenon timely as failure to recognize this phenomenon or delayed treatment can lead to catastrophic results, as in the case presented above.

## References

[1] Farkas JD, Long B, Koyfman A, Menson K (2020). BRASH syndrome: bradycardia, renal failure, AV blockade, shock, and hyperkalemia. J Emerg Med.

[2] Hayes DF, Werner MH, Rosenberg IK, Lucas CE, Westreich M, Bradley V (1974). Effects of traumatic hypovolemic shock on renal function. J Surg Res.

[3] Momoniat T, Ilyas D, Bhandari S (2019). ACE inhibitors and ARBs: managing potassium and renal function. Cleve Clin J Med.

[4] Noble K, Isles C (2006). Hyperkalaemia causing profound bradycardia. Heart.

[5] Da Costa D, Brady WJ, Edhouse J (2002). Bradycardias and atrioventricular conduction block. BMJ.

[6] Kunis CL, Charney AN (1981). Potassium and renal failure. Compr Ther.

[7] Raebel MA (2012). Hyperkalemia associated with use of angiotensin-converting enzyme inhibitors and angiotensin receptor blockers. Cardiovasc Ther.

[8] Horisberger JD, Giebisch G (1987). Potassium-sparing diuretics. Ren Physiol.

[9] Makris K, Spanou L (2016). Acute kidney injury: definition, pathophysiology and clinical phenotypes. Clin Biochem Rev.

[10] Hahn L, Hahn M (2015). Carvedilol-induced hyperkalemia in a patient with chronic kidney disease. J Pharm Pract.

[11] Aoun M, Tabbah R (2016). Case report: severe bradycardia, a reversible cause of "Cardio-Renal-Cerebral Syndrome". BMC Nephrol.

[12] Kosaraju A, Pendela VS, Hai O (2021). Cardiogenic Shock. StatPearls [Internet].

[13] Sattar Y, Bareeqa SB, Rauf H, Ullah W, Alraies MC (2020). Bradycardia, renal failure, atrioventricular-nodal blocker, shock, and hyperkalemia syndrome diagnosis and literature review. Cureus.

[14] Dépret F, Peacock WF, Liu KD, Rafique Z, Rossignol P, Legrand M (2019). Management of hyperkalemia in the acutely ill patient. Ann Intensive Care.

[15] Sidhu S, Marine JE (2020). Evaluating and managing bradycardia. Trends Cardiovasc Med.

[16] Littmann L, Gibbs MA (2018). Electrocardiographic manifestations of severe hyperkalemia. J Electrocardiol.

